# Left ventricular dysfunction in pulmonary arterial hypertension is attributed to underfilling rather than intrinsic myocardial disease: a CMR 2D phase contrast study

**DOI:** 10.1038/s41598-024-68254-5

**Published:** 2024-07-27

**Authors:** Ashwin Venkateshvaran, Jessica Bohlin, Barbro Kjellström, Elsa Bergström, Anders Nelsson, Anna Werther Evaldsson, Göran Rådegran, Håkan Arheden, Ellen Ostenfeld

**Affiliations:** 1 Clinical Physiology, Department of Clinical Sciences Lund, Skåne University Hospital, Lund University, Lund, Sweden; 2 Department of Cardiology, Skåne University Hospital, Malmö, Sweden; 3 Department of Clinical Sciences Lund, Cardiology, and the Section for Heart Failure and Valvular Disease, Skåne University Hospital, Lund University, Lund, Sweden

**Keywords:** Heart failure, Pulmonary arterial hypertension, Cardiac magnetic resonance imaging, Chronic obstructive pulmonary disease, Biophysics

## Abstract

The pathophysiology underlying impaired LV function in PAH remains unclear, with some studies implicating intrinsic myocardial dysfunction and others pointing to LV underfilling. Evaluation of pulmonary vein area (PVA) and flow may offer novel, mechanistic insight by distinguishing elevated LV filling pressure common in myocardial dysfunction from LV underfilling. This study aimed to elucidate LV filling physiology in PAH by assessing PVA and flow using cardiac magnetic resonance (CMR) and compare pulmonary vein flow in PAH with HFrEF as a model representing elevated filling pressures, in addition to healthy controls. Patients with PAH or heart failure with reduced ejection fraction (HFrEF) referred for CMR were retrospectively reviewed, and healthy controls were included as reference. Pulmonary vein S, D and A-wave were compared between groups. Associations between pulmonary vein area (PVA) by CMR and echocardiographic indices of LV filling pressure were evaluated. Nineteen patients with PAH, 25 with HFrEF and 24 controls were included. Both PAH and HFrEF had lower ejection fraction and S-wave velocity than controls. PAH displayed smaller LV end-diastolic volumes than controls, while HFrEF demonstrated larger PVA and higher A-wave reversal. PVA was associated with mitral E/e′ ratio (r^2^ = 0.10; *p* = 0.03), e′ velocity (r^2^ = 0.23; *p* = 0.001) and left atrial volume (r^2^ = 0.07; *p* = 0.005). Among PAH, PVA was not associated with LV-GLS. A PVA cut-off of 2.3cm^2^ displayed 87% sensitivity and 72% specificity to differentiate HFrEF and PAH (AUC = 0.82). PAH displayed lower pulmonary vein S-wave velocity, smaller LV volume and reduced function compared with controls. Reduced LV function in PAH may be owing to underfilling rather than intrinsic myocardial disease. PVA demonstrates promise as a novel, non-invasive imaging marker to assess LV filling status.

## Introduction

Pulmonary arterial hypertension (PAH) is a rare but potentially fatal disorder characterized by pulmonary vascular remodelling leading to right ventricular (RV) pressure overload and progressive RV dysfunction. Although commonly considered a right-heart disease, impaired left ventricular (LV) global longitudinal strain and reduced stroke volume are frequently observed in PAH and associated with poor prognosis^1^. However, the pathophysiology underlying impaired LV function in PAH is unclear.

LV dysfunction in PAH has been ascribed to intrinsic myocardial dysfunction, indicated by significant reductions in cross-sectional area and maximal force-generating capacity of cardiomyocytes in the LV myocardium^2^. However, as seen in heart failure with reduced ejection fraction (HFrEF), intrinsic myocardial dysfunction results in elevated left-sided filling pressures. Other studies have attributed LV dysfunction in PAH to left-sided underfilling secondary to elevated pulmonary vascular resistance^3^. While both PAH and HFrEF demonstrate reduced stroke volumes and are considered low-flow hemodynamic phenotypes, they exhibit different LV filling profiles. Novel imaging approaches targeted at clarifying mechanistic aspects of impaired LV pump function in PAH may improve early disease identification and targeted therapy.

The pulmonary veins act as a conduit for left-sided filling and could potentially distinguish LV underfilling from intrinsic LV myocardial dysfunction. Structural and flow-related alterations of the pulmonary veins are direct consequences of elevated LV filling pressures but are challenging to interrogate using echocardiography^4^. Cardiac magnetic resonance (CMR) imaging is ideally suited for pulmonary vein assessment as it is unrestricted by acoustic windows and displays superior spatial resolution. Additionally, 2D phase contrast imaging provides reproducible flow measurements independent of anatomical orientation^5^.

The aim of this study was to investigate if patients with PAH displayed altered LV filling physiology by assessing pulmonary vein area (PVA) and flow patterns using CMR, with HFrEF as a model of elevated filling pressures in addition to healthy controls.

## Methods

### Study population

Adult subjects diagnosed with either PAH or clinician judged HFrEF with EF < 40% that underwent a clinically indicated CMR at the Skåne University Hospital, Lund, between 2016 and 2021 were retrospectively reviewed. Exclusion criteria were atrial fibrillation, more than mild mitral or aortic valve disease, poor image quality, more than 14 days and/or changes in medication or clinical status between CMR and echocardiography. Controls were included from a database of healthy volunteers with no known cardiovascular co-morbidities or medications, and with normal systemic blood pressure, ECG, CMR and echocardiographic examinations. The study was approved by the Regional Ethical Review Board of Lund (EPN 2010-248, EPN 2013-891, EPN 2013-900, EPN 2015-248, EPN 2017-829, EPN 2018-048, EPN 2004-741). All participants provided written informed consent. The study complies with the Declaration of Helsinki and is in accordance with the STROBE checklist for observational studies.

### CMR

**Figure 1:**
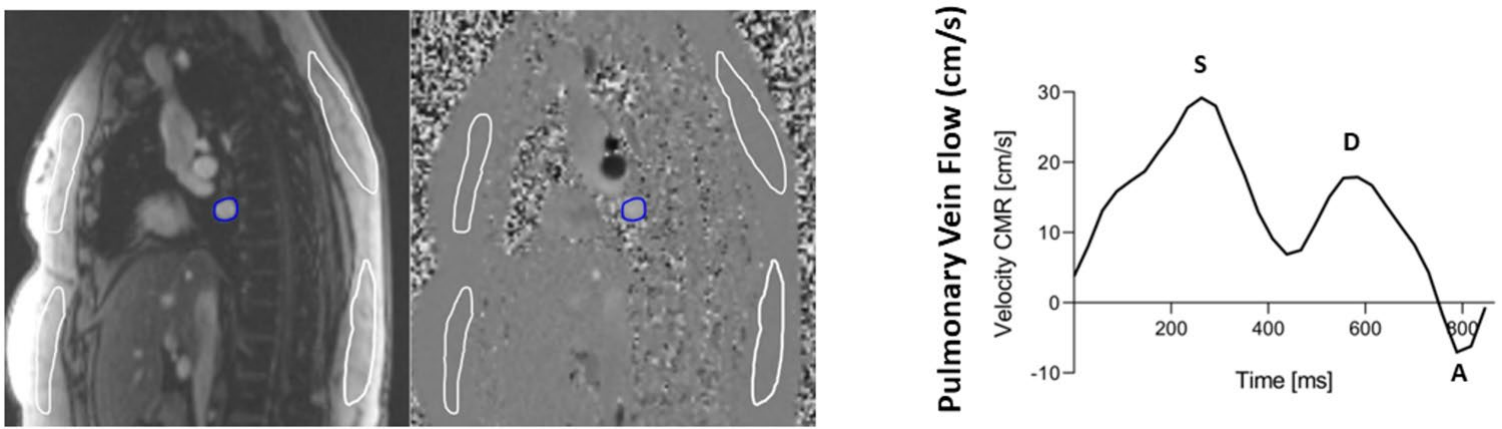
Magnitude and phase images from magnetic resonance imaging examination and the corresponding flow curves in the pulmonary vein in a patient with pulmonary arterial hypertension. Time velocity curves of the right lower pulmonary vein (blue) showing systolic (S), Diastolic (D) and the atrial reversal (A) flows.

CMR studies were performed using standard protocols on a commercially available 1.5 T scanner (MAGNETOM Aera, Siemens Healthcare, Erlangen, Germany). Standard cine balanced steady-state free precession images of long-axis and a short-axis stacks were acquired in a supine, head-first position with ECG-triggering and during end-respiratory breath-hold. Phase-contrast imaging was performed with the imaging plane positioned perpendicular to blood flow of the ascending aorta, main pulmonary artery, and the right pulmonary vein. Velocity encoding was recorded using a velocity of 200 cm/sec for the aorta and 150 cm/sec for the pulmonary artery. PV anatomy was defined using magnitude images from flow assessment acquired according to international recommendations in axial and double oblique planes^6^. PVA was delineated across all frames during the cardiac cycle and maximal PVA was selected. Velocity encoding was recorded using a velocity of 80 cm/sec for the pulmonary vein. Pulmonary vein images with flow acquisition in close proximity of the left atrium or distally to branching of the veins were excluded from the analysis. All images were analyzed in the freely available software Segment v 4.0 R11044b (Medviso, Lund, Sweden)^7^. Quantification of flow wave forms was performed by delineating the pulmonary vein of interest in all cardiac time phases and calculating net forward blood flow. Pulmonary flow maximal systolic (S), diastolic (D) and atrial reversal (A) velocities were registered. (Fig. 1) Maximal PVA was assessed across all time phases. LV global longitudinal strain (LV-GLS) was analyzed in the 4-chamber view using feature tracking in controls and PAH cohort. The endo- and epicardial borders were manually delineated in the end-diastolic frame, and the software automatically tracked movement during the cycle using a vector-based algorithm.

### Echocardiography

Patients underwent a standard echocardiogram in keeping with current guidelines^8^ employing commercial ultrasound systems (Vivid E9, General Electric, Horten, Norway; Philips iE33, Eindhoven, Netherlands and Acuson SC2000, Siemens Healthcare, Erlangen, Germany) equipped with an adult matrix-array transducer. 2D gray-scale images were acquired at 50–80 frames/sec over 3 cardiac cycles. Doppler tracings were recorded using a sweep speed of 100 mm/sec. Echocardiographic variables that correspond with LV diastolic function, i.e., mitral E and A wave velocity, mitral e′ velocity, E/e′ ratio, tricuspid valve regurgitation (TR) velocity and indexed left atrial volume (LAVI) were registered. All images were subsequently exported and analyzed offline by an experienced investigator (AV) blinded to CMR data.

### Inter- and intraobserver variability

To evaluate the intra- and inter-observer variability of the methods, PVA, S-, D- and A-wave velocity were measured 4 weeks apart in 10 subjects by the same investigator (JB), and by two different investigators (JB, EB).

### Statistical methods

Continuous variables were expressed as median and interquartile range according to distribution, after normality was visually dismissed using QQ plots. Categorical variables were expressed as numbers and proportion in percentage. PAH, HFrEF and controls were compared using the Kruskal–Wallis test, and pair-wise comparisons using the post-hoc Dunn test. The coefficient of determination (r^2^) was used to report proportion of the variation in PVA that is predicted from echocardiographic measures of LV function. A posthoc power calculation was conducted to determine the sample size required to detect a clinically significant difference between patients with PAH and controls, using an alpha of 0.05 and a power of 80%. The effect size was set to correspond to a mean PVA of 2.5 cm^2^ in PAH patients (equivalent to the mean PVA in controls ± 1 SD), resulting in a required sample size of 16 participants per group. Intraclass correlation coefficient (ICC) and bias according to Bland–Altman was used to assess intra- and inter-observer variability^9^. ICC was predefined as poor (< 0.5), adequate (0.5–0.74), good (0.75–0.89), or excellent (≥ 0.90). Receiver operator characteristics (ROC) analysis and Youden index was employed to determine the discriminatory ability of PVA and cut-off to differentiate HFrEF from PAH. Tests were performed at 95% confidence intervals and a *p*-value < 0.05 was considered statistically significant. IBM SPSS statistics version 23.0 was employed for all statistical analysis.

### Ethics approval

The study was approved by the Regional Ethical Review Board of Lund (EPN 2010-248, EPN 2013-891, EPN 2013-900, EPN 2015-248, EPN 2017-829, EPN 2018-048, EPN 2004-741). All participants provided written informed consent. The study complies with the Declaration of Helsinki and is in accordance with the STROBE checklist for observational studies.

## Results

A retrospective review was conducted on 83 subjects of which 15 were excluded, due to atrial fibrillation (n = 5), suboptimal positioning of the pulmonary vein interrogation plane (n = 3) or absence of pulmonary vein phase contrast images (n = 7). In effect, 68 subjects were included in the final analysis. This included 24 healthy controls, 25 patients with HFrEF and 19 patients with PAH.

## Patient characteristics

Clinical characteristics as well as CMR and echocardiographic data are presented in Table 1. Both patient groups demonstrated lower LVEF compared with controls although the PAH group was within normal limits. PAH patients demonstrated lower LV end-diastolic volumes (LVEDV), LV stroke volumes, LV-GLS, mitral E/A ratios and septal e′ velocities, higher mitral A-wave velocities, and TR max velocities when compared with controls. HFrEF demonstrated larger LVEDV and end-systolic volumes (LVESV), left atrial volumes, mitral E/e′ ratio, and lower mitral e′ velocity and higher body surface area compared with controls.

**Table 1:** Clinical characteristics, magnetic resonance imaging (CMR) and echocardiographic data for healthy controls, HFrEF and PAH.

	Control (n = 24)	HFrEF (n = 25)	PAH (n = 19)	*P* value
*Demographics*				
Age (years)	64 (61–66)	66 (60–74)	69 (55–74)	0.33
Women	15 (63%)	7 (28%)	15 (79%)	0.002
BMI (kg/m^2^)	25 (22–26.5)	28 (25–29)	26 (23–30)	0.06
BSA (m^2^)	1.8 (1.7–2.0)	2.0 (1.8–2.1)*	1.9 (1.7–2.0)^##^	0.01
*CMR and 2D phase contrast*				
LV end diastolic volume (ml)	152 (135–175)	286 (220–389)***	118 (95–148)*^###^	< 0.001
LV end systolic volume (ml)	65 (48–74)	185 (154–290)***	60 (48–76)^###^	< 0.001
LV stroke volume (ml)	91 (79–98)	81 (68–107)	59 (52–76)***^##^	0.002
LV ejection fraction (%)	60 (55–63)	30 (21–35)*	53 (45–58)*	< 0.001
Pulmonary vein area (cm^2^)	1.7 (0.7–2.5)	2.9 (0.8–3.8)***	2.0 (0.7–2.6)^###^	< 0.001
Pulmonary vein S wave velocity (cm/s)	48 (42–55)	40 (28–49)*	41 (29–48)*	0.03
Pulmonary vein D wave velocity (cm/s)	28 (24–34)	32 (26–60)	35 (26–42)	0.13
Pulmonary vein A wave velocity (cm/s)	15 (13–18)	25 (14–37)**	14 (11–17)^#^	0.01
Global longitudinal strain (%)	− 22 (− 20 to − 24)	− 9 (− 7 to − 12)***	− 18 (− 16 to − 21)**^###^	< 0.001
*Echocardiography*				
Mitral E wave velocity (cm/s)	68 (48–79)	69 (49–94)	55 (39–70)	0.24
Mitral A wave velocity (cm/s)	61 (54–77)	82 (44–106)	77 (58–112)*	0.09
Mitral E/A ratio	0.99 (0.79–1.3)	0.77 (0.56–1.82)	0.67 (0.62–0.73)***	0.007
Mitral septal e′ velocity (cm/s)	8.6 (8–9.3)	4.9 (4.3–5.8)***	6.2 (3.9–7.7)*	< 0.001
Mitral lateral e′ velocity (cm/s)	10.9 (10–12)	7.3 (6.1–8)***	8.7 (7.4–14.9)	< 0.001
Mitral average e′ velocity (cm/s)	10 (9.2–11)	6.2 (5.3–6,6)***	7.5 (5.8–10.6)	< 0.001
Mitral average E/e′ ratio	6.3 (5.6–7.5)	10.8 (7.8–14.7)**	6.5 (5.3–9.2)^#^	< 0.001
TR max velocity (m/s)	2.2 (0.5–2.3)	2.4 (2.4–2.9)	4.0 (3.6–4.3)***^###^	< 0.001
LA volume index (ml/m^2^)	21 (15–29)	36 (28–48)***	21 (17–28)^##^	< 0.001

Data presented as median (Q1; Q3) or number (%). HFrEF = heart failure with reduced ejection fraction, PAH = pulmonary arterial hypertension, BMI = body mass index, BSA = body surface area, S wave = peak systolic velocity in pulmonary vein, D wave = peak diastolic velocity in pulmonary vein, A wave = peak atrial reversal velocity in pulmonary vein, E wave velocity = peak velocity from left ventricular relaxation in early diastole, A wave velocity = peak velocity in late diastole due to atrial contraction, E/A ratio = ratio E wave to A wave, e′ = wave of early diastolic mitral valve annular velocity, TR max velocity = tricuspid regurgitation max velocity, LA = left atrial.

*p < 0.05, ** < 0.01, *** < 0.001 compared to healthy controls.

^#^*p* < 0.05, ## < 0.01, ### < 0.001 compared to HFrEF.

### Pulmonary vein characteristics

**Figure 2:**
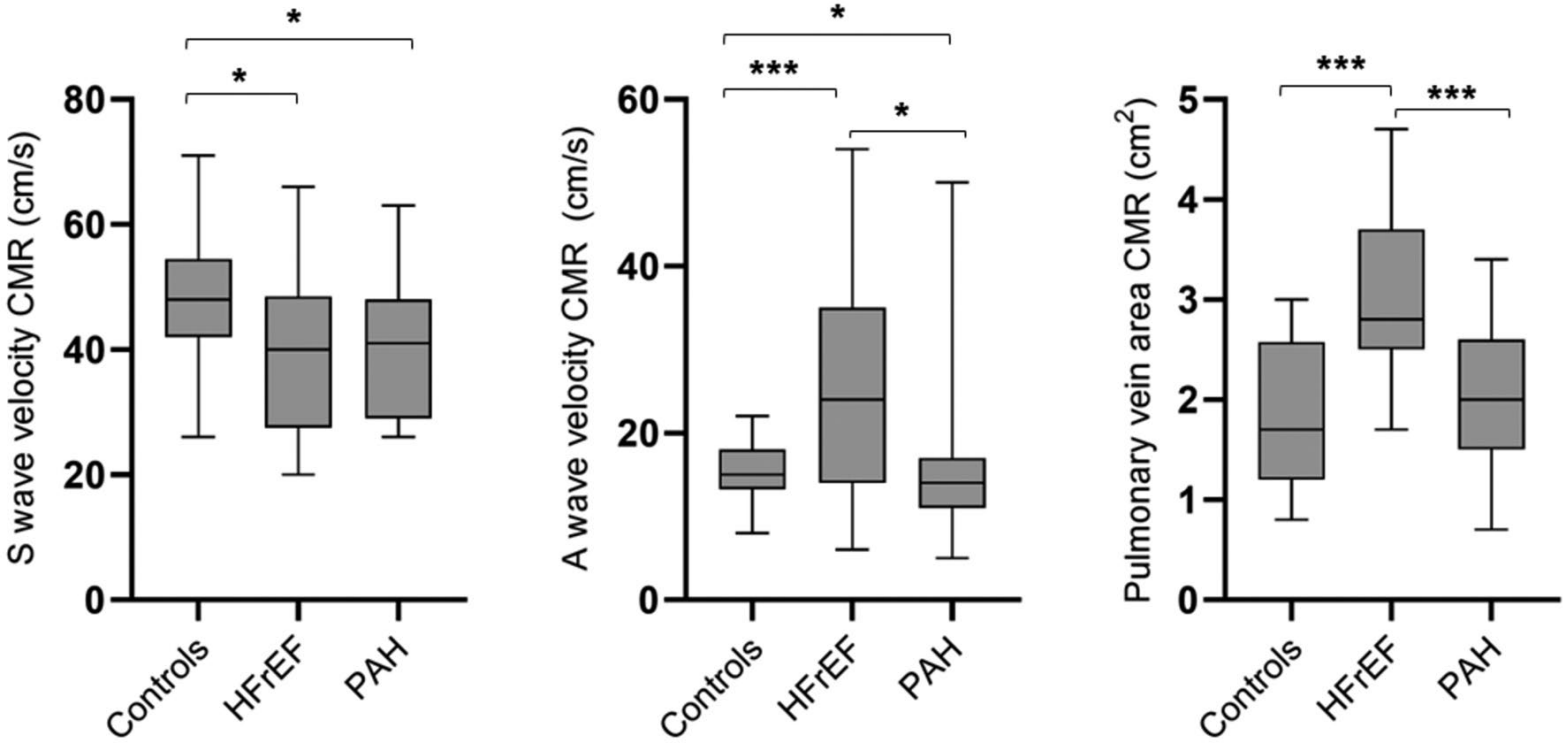
Box plots comparing pulmonary vein characteristics obtained using cardiac magnetic resonance imaging (CMR) between controls, heart failure with reduced ejection (HFrEF) and pulmonary arterial hypertension (PAH). *p < 0.05, ** < 0.01, *** < 0.001.

Evaluation of PVA, S-, D- and A-wave velocity by CMR was feasible in 97%, 100%, 100% and 97% of subjects respectively. Both HFrEF and PAH demonstrated lower S-wave velocity when compared with controls. HFrEF demonstrated larger PVA when compared with controls and PAH. In addition, HFrEF demonstrated larger A-wave reversal compared with controls and PAH (Fig. 2, Table 1).

### Associations between PVA and LV size and function

**Figure 3:**
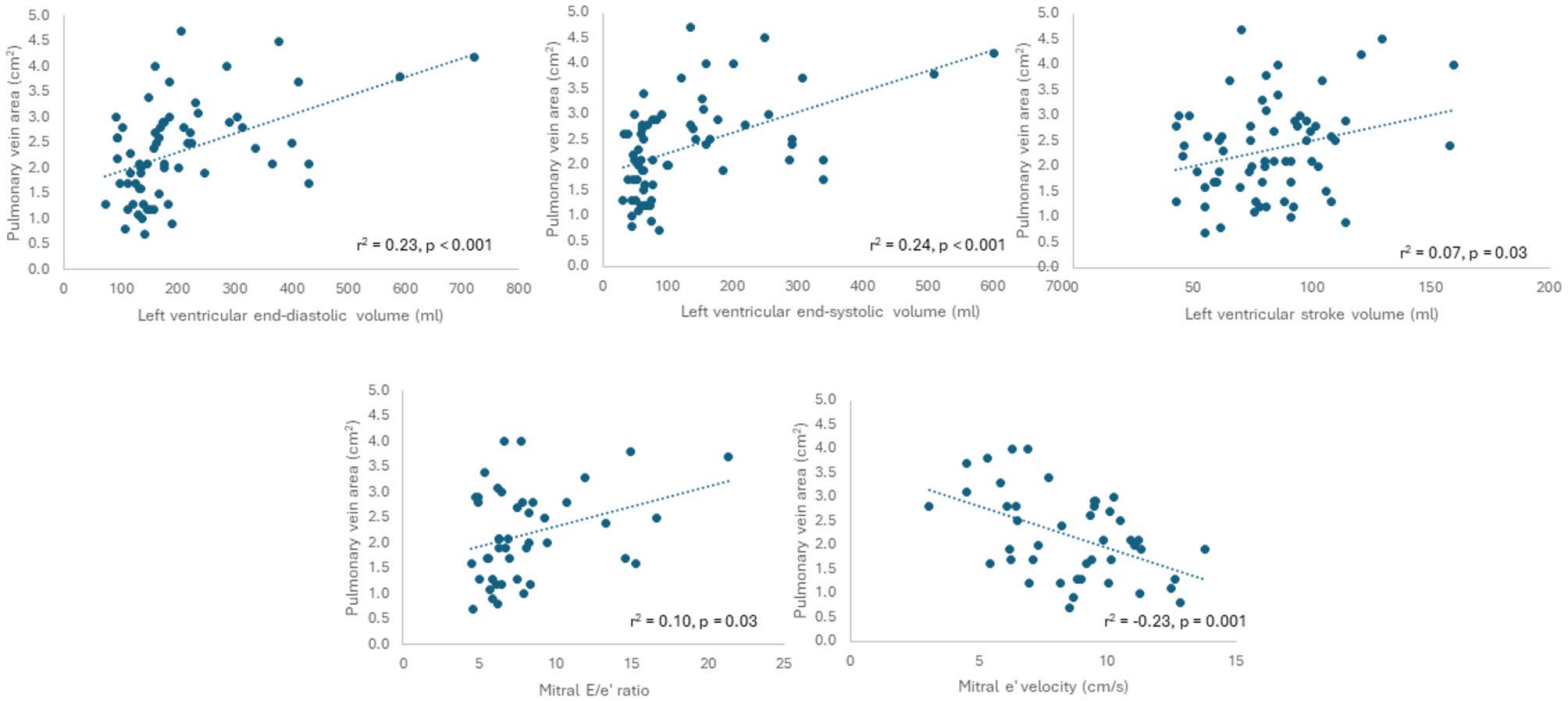
Scatter plots displaying correlations between pulmonary vein area and LV functional parameters.

PVA was associated with LV end-diastolic volumes (LVEDV) (r^2^ = 0.23, *p* < 0.001), LV end-systolic volumes (LVESV) (r^2^ = 0.24, *p* < 0.001) and LV stroke volume (r^2^ = 0.07, *p* = 0.03). Furthermore, PVA was associated with echocardiographic diastolic indices such as mitral E/e′ ratio (r^2^ = 0.10; *p* = 0.03), mitral e′ velocity (r^2^ = 0.23; *p* = 0.001) and LAVI (r^2^ = 0.16; *p* = 0.005) (Fig. 3). No significant associations were observed between PVA and LAVI, mitral E/e′ ratio or mitral e′ velocity when subgroups were separately considered (Supplemental Table 1). PVA was associated with LV-GLS in the total population (r^2^ = 0.32, *p* < 0.001). No significant associations were found between PVA and LV-GLS in the PAH cohort.

### PVA as a distinguisher of filling phenotypes

**Figure 4:**
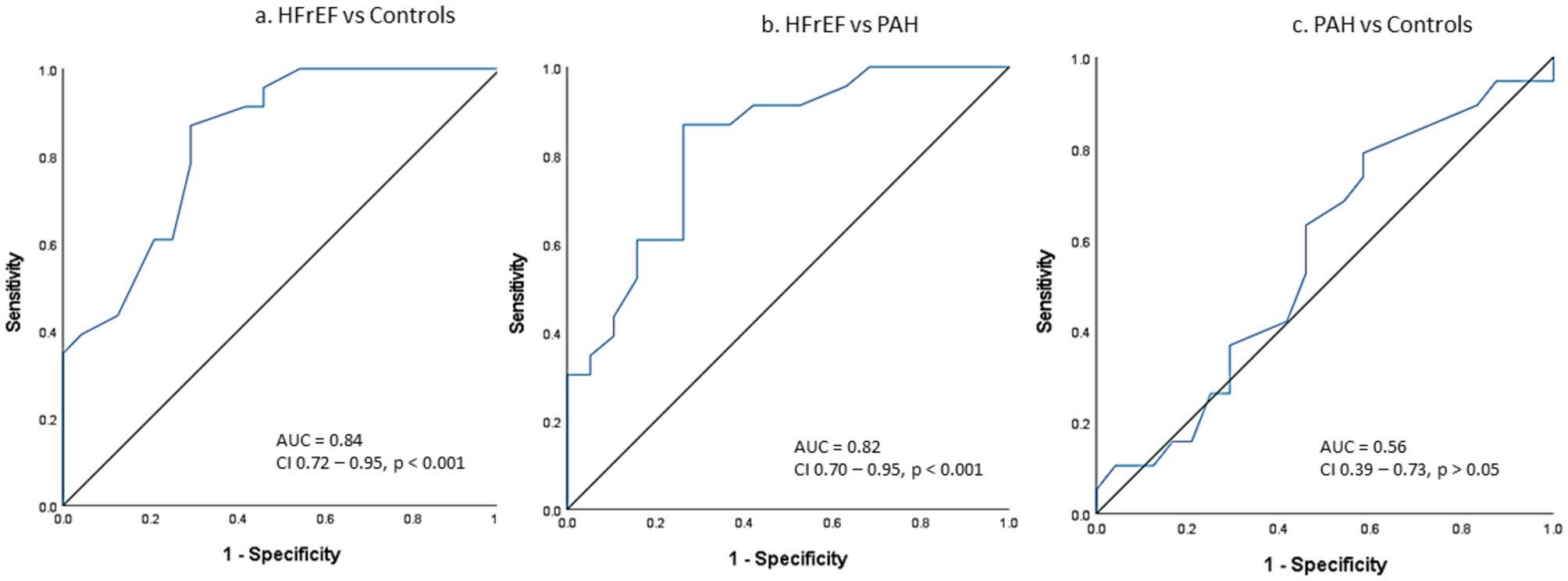
ROC analysis demonstrating ability of pulmonary vein area (PVA) to differentiate subgroups within the cohort.

PVA demonstrated ability to differentiate HFrEF from controls (AUC = 0.84; CI 0.72–0.95; *p* < 0.001) (Fig. 4a) and from PAH (AUC = 0.82; CI 0.70–0.95; *p* < 0.001) (Fig. 4b). At a Youden cut-off value of 2.3 cm^2^, PVA displayed 87% sensitivity and 72% specificity to differentiate HFrEF from PAH. However, PVA did not differentiate PAH from controls (AUC = 0.56; CI 0.39–0.73 *p* > 0.05) (Fig. 4c).

In comparison, mitral E/e′ displayed lower diagnostic performance (AUC = 0.76 vs 0.84, *p* = 0.03), LAVI displayed comparable performance (AUC = 0.87) and mitral e′ displayed a trend to significant diagnostic performance (AUC = 0.76, *p* = 0.051).

### Intra- and interobserver agreement of pulmonary vein assessment by CMR

PVA assessed by CMR demonstrated excellent intra- (ICC = 0.98; Bias =  − 0.17 ± 0.26 cm^2^) and interobserver agreement (ICC = 0.96; Bias =  − 0.17 ± 0.26 cm^2^), suggesting high measurement reproducibility. Excellent intraobserver agreement was observed for S-wave (ICC = 0.91; Bias = 1.3 ± 6.0 cm/s), D-wave (ICC = 0.98; Bias =  − 2.3 ± 4.5 cm/s) and A-wave velocities (ICC = 0.92; Bias = 0.8 ± 3.8 cm/s). Also, excellent interobserver agreement was observed for S-wave (ICC = 0.98; Bias = 0.40 ± 1.9 cm/s), D-wave (ICC = 0.90; Bias = 2.4 ± 7.6 cm/s) and A-wave velocities (ICC = 0.95; Bias = 0.7 ± 3.0 cm/s).

## Discussion

In this study, pulmonary vein size and flow assessed using CMR provides mechanistic evidence to support LV underfilling as the prevailing contributing factor to reduced LV pump performance in PAH. Both patient groups, PAH and HFrEF, demonstrated reduced LV EF and alterations in pulmonary venous return when compared with healthy controls. However, PAH demonstrated reduced LVEDV compared with controls, and PVA was not associated with LV-GLS in this subcohort. HFrEF displayed distended pulmonary veins, higher E/e′ ratio and increased backward flow velocities during atrial contraction. Further, PVA was associated with LV filling pressures estimated by echocardiography.

Both HFrEF and PAH demonstrated lower pulmonary vein S-wave velocities than controls in this study. In HFrEF, reduced S-wave velocities has previously been attributed to decreased systolic descent of the mitral annulus^10^ and lowered LV contractility^11^. PAH, however, is characterized by normal left-sided filling pressures and isolated elevation of pulmonary arterial pressures^12^. Studies suggest that pulmonary vein S-wave velocity propagation is influenced both by LA suction and reduced forward flow across the pulmonary bed^13,14^. An earlier study employing CMR suggests that reduced LV-GLS in PAH was associated with reduced stroke volumes in addition to small LV & LA volumes^3^. In the present study, a reduced S-wave velocity together with reduced LVEDV may simply reflect reduced preload secondary to increased pulmonary vascular resistance and/or impaired RV function^12^. This is also supported by the lower LV-GLS in the PAH group when compared with controls in our study.

In the present study, we also demonstrate higher A-wave velocities in the HFrEF group compared with controls and PAH. Mathematical models of the pulmonary veins suggest that wave flow propagation originates from the LA and is transmitted backwards to the pulmonary bed, generating wave reflections owing to vessel impedance and pulmonary vascular bed compliance^15^. The presence of increased LV filling pressures in HFrEF may explain the increased pulmonary vein A-wave velocities in the present study and lead to distended PVA in the HFrEF group. An important finding in this study is the association of PVA with multiple echocardiographic indices of LV filling pressures. Earlier CMR studies suggest that increased LA volumes demonstrate high sensitivity and specificity to identify HFrEF^16^. Our findings extend these findings to suggest that PVA size reflects LV filling status over LA mechanical stretch, and may augment conventional evaluation of left-sided filling pressures. Current echocardiographic recommendations employ assessment of inferior vena cava to estimate central venous pressure^8^. The utility and accuracy of PVA to estimate and grade left-sided filling pressures needs to be explored in studies employing right heart catheterization performed simultaneously with CMR.

Another observation in this study is the high feasibility of CMR to characterize pulmonary vein flow. While A-wave duration provides important information on the status of elevated filling pressures, it cannot be solely relied upon owing to uninterpretable flow profiles in as many as 35–42% of transthoracic echocardiography evaluations^4,17^. Contrast echocardiography improves feasibility and accuracy of data interpretation^18^, but is minimally invasive and involves injection of ultrasound enhancing agents. Our study demonstrates that CMR provides highly feasible, reproducible measurements of pulmonary vein S, D and A wave without the need for intravenous contrast.

Lower velocities on 2D phase contrast CMR when compared with Doppler echocardiography are also in keeping with previous studies^19^. While Doppler employs ultrasonic frequency shifts to assess blood flow velocities, 2D phase contrast detects phase shift of water molecules under a gradient magnetic field. In addition, 2D phase contrast imaging displays relatively lower temporal resolution than echocardiography, and this may impact velocity measurements. Nevertheless, feasibility was high, and CMR overcomes the limitations of acoustic windows and body habitus that result in generally poorer lung vein interrogation by echocardiography. In addition to highly reproducible PVA and PV flow measurements, CMR offers reference standard measurements of LV mass and volume, LA volume and provides detailed myocardial tissue characterization, providing strong complementary role in diastolic dysfunction assessment. Recent studies suggest that CMR can be utilized to evaluate grades of diastolic dysfunction and demonstrates strong agreement with echocardiography^20^. Our study suggests that assessment of PVA may offer incremental value to this assessment, but its accuracy needs to be validated against reference standard invasive measurements of filling pressures in future studies.

The results of our physiological study are hypothesis-generating and suggest that PVA offers promise as a novel diagnostic marker to distinguish LV filling states. Extension of this finding to explore its utility to distinguish Group 1 (PAH) from Group 2 PH (due to left heart disease) as per current recommendations^21^ requires validation in future studies with reference standard invasive pressure assessment. Such studies will also permit exploration of invasive correlates of PVA and establish its clinical utility as a diagnostic distinguisher for disease stratification.

Some limitations of the current study should be noted. Firstly, the absence of right heart catheterization data as reference, secondly its retrospective design, and thirdly the cross-sectional nature of the study which limits any conclusions concerning causality. 

## Conclusions

PAH displayed smaller LV volumes, reduced PV S wave velocity and reduced function compared with controls. Among PAH, PVA displayed no association with myocardial contractility represented by LV-GLS. Reduced LV function in PAH may be owing to underfilling rather than intrinsic myocardial disease. PVA demonstrates promise as a novel, non-invasive imaging marker to assess LV filling status.

### Supplementary Information

Supplementary Information.

## Data Availability

The datasets used and/or analysed during the current study are available from the corresponding author upon reasonable request.
