# Supplementary material for: Left ventricular dysfunction in pulmonary arterial hypertension is attributed to underfilling rather than intrinsic myocardial disease: a CMR 2D phase contrast study
Source: Sci Rep. 2024 Jul 27;14:17280. doi: 10.1038/s41598-024-68254-5 (PMC11283488; doi:10.1038/s41598-024-68254-5)

**Supplemental Table 1.** Correlations between pulmonary vein area and echocardiographic indices of diastolic dysfunction in cohort subgroups

|  | **Mitral E/e’ ratio** | | **Mitral e’ velocity** | | **LAVI** | |
| --- | --- | --- | --- | --- | --- | --- |
|  | r-value | p-value | r-value | p-value | r-value | p-value |
| Controls | -0.01 | 0.94 | -0.09 | 0.66 | -0.15 | 0.46 |
| HFrEF | -0.05 | 0.86 | -0.32 | 0.31 | 0.36 | 0.23 |
| PAH | -0.03 | 0.92 | -0.24 | 0.55 | 0.19 | 0.55 |

HFrEF = heart failure with reduced ejection fraction, PAH = pulmonary arterial hypertension, LAVI = left atrial volume index


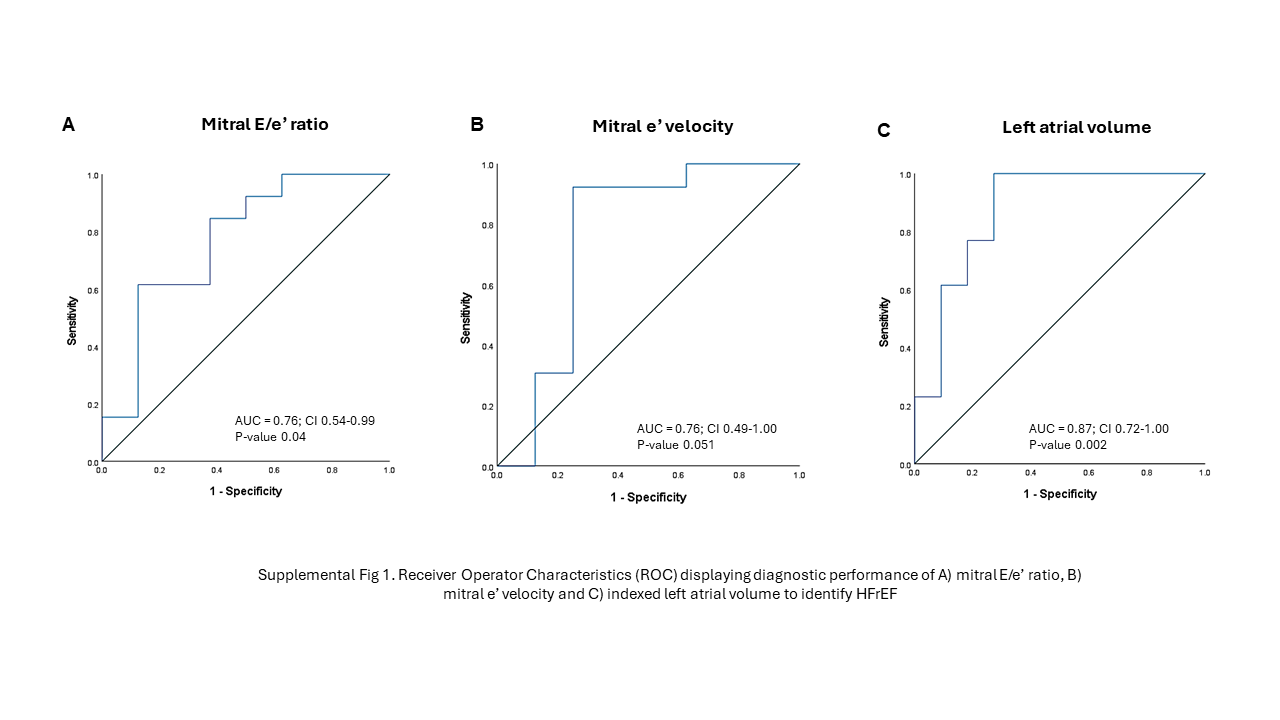

Supplement: Supplementary file 1 — Supplementary Information. [file 41598_2024_68254_MOESM1_ESM.docx]
